# HLA, gut microbiome and hepatic autoimmunity

**DOI:** 10.3389/fimmu.2022.980768

**Published:** 2022-08-18

**Authors:** Benedetta Terziroli Beretta-Piccoli, Giorgina Mieli-Vergani, Diego Vergani

**Affiliations:** ^1^ Faculty of Biomedical Sciences, Epatocentro Ticino and Università della Svizzera Italiana, Lugano, Switzerland; ^2^ MowatLabs, Faculty of Life Sciences and Medicine, King’s College London, King’s College Hospital, London, United Kingdom

**Keywords:** autoimmune hepatitis, AIH, primary biliary cholangitis (PBC), primary sclerosing cholangites (PSC), gut microbiome, human leucocyte antigen (HLA)

## Abstract

Genetic susceptibility to autoimmune liver diseases is conferred mainly by polymorphisms of genes encoding for the human leukocyte antigens (HLA). The strongest predisposition to autoimmune hepatitis type 1 (AIH-1) is linked to the allele DRB1*03:01, possession of which is associated with earlier disease onset and more severe course. In populations where this allele is very rare, such as in Asia, and in DRB1*03-negative patients, risk of AIH-1 is conferred by DRB1*04, which is associated with later disease onset and milder phenotype. AIH type 2 (AIH-2) is associated with DRB1*07. The pediatric condition referred to as autoimmune sclerosing cholangitis (ASC), is associated with the DRB1*13 in populations of Northern European ancestry. DRB1*1501 is protective from AIH-1, AIH-2 and ASC in Northern European populations. Possession of the DRB1*08 allele is associated with an increased risk of primary biliary cholangitis (PBC) across different populations. DRB1*03:01 and B*08:01 confer susceptibility to primary sclerosing cholangitis (PSC), as well as DRB1*13 and DRB1*15 in Europe. The hepatic blood supply is largely derived from the splanchnic circulation, suggesting a pathophysiological role of the gut microbiome. AIH appears to be associated with dysbiosis, increased gut permeability, and translocation of intestinal microbial products into the circulation; molecular mimicry between microbial and host antigens may trigger an autoaggressive response in genetically-predisposed individuals. In PBC an altered enteric microbiome may affect intestinal motility, immunological function and bile secretion. Patients with PSC have a gut microbial profile different from health as well as from patients with inflammatory bowel disease without PSC.

## Introduction

Autoimmune liver diseases, including autoimmune hepatitis (AIH), primary biliary cholangitis (PBC) and primary sclerosing cholangitis (PSC) are believed to be triggered by as yet poorly characterized environmental factors that lead to loss of tolerance to self-antigens in genetically predisposed individuals. Among genetic factors, the most relevant lie within the human leukocyte antigen (HLA) region on chromosome 6. Among environmental factors, infections, drug/toxins and microbiome have been suggested to be involved. A recent animal model of AIH, using transgenic mice on the non-obese diabetic (NOD) background carrying human HLA-DR3 and immunized with a DNA plasmid coding for a fusion protein of human CYP2D6/FTCD – the autoantigens targeted in AIH type 2 -, showed

that not only did the human HLA-DR3 predispose the animals to AIH, but also influenced the microbiome composition compared to the HLA-DR3 negative controls (1), suggesting a direct link between HLA and microbiome. The present short review recapitulates current knowledge on HLA associations and microbiome changes in autoimmune liver disease.

## The human leukocyte antigen

HLA are proteins expressed on the surface of a variety of cell types involved in antigen recognition by T cells. HLA molecules physiologically create a groove which embeds a short antigenic peptide to be presented to the T cell receptor. The genes encoding for the HLA proteins, located on the short arm of chromosome 6, are highly polymorphic, ensuring that different individuals are able to recognize and respond to a wide range of different antigenic peptides. Genetic predisposition to autoimmune diseases is often conferred by HLA alleles, and autoimmune liver diseases are no exception. Class I HLA proteins, expressed on all nucleated cells and recognized by CD8 T cells, display peptides derived from cytosolic proteins; class II HLA molecules, expressed on B cells, dendritic cells and macrophages, and recognized by CD4 T cells, display peptides derived from extracellular proteins, upon endocytosis. The HLA genes are characterized also by linkage disequilibrium extending over a large number of genes, meaning that certain alleles are inherited together with higher frequency than expected by random recombination during meiosis. This feature may make it difficult to assess the impact of a specific allele on the pathophysiology of diseases associated with HLA alleles. Interestingly, the so called 8.1 ancestral genotype is a very long haplotype containing alleles predisposing to AIH and PSC, as well as to a host of autoimmune diseases in Caucasian populations, including myositis syndromes, Sjögren syndrome, myasthenia gravis, and celiac disease (2, 3). The clinical observation of the frequent coexistence of hepatic and extrahepatic autoimmunity is possibly linked to a shared genetic predisposition involving different autoantigens.

### Autoimmune hepatitis

AIH is a rare chronic inflammatory liver disease affecting all ages, characterized by positive autoantibodies, elevated immunoglobulin G (IgG) serum levels, interface hepatitis at liver histology, and swift response to immunosuppressive treatment (4). Some 10-20% of AIH patients respond unsatisfactorily to pharmacologic treatment and progress to liver failure, requiring liver transplantation (5). AIH is subdivided into type 1 (AIH-1) and type 2 (AIH-2) according to autoimmune serology, AIH-1 being associated with positive anti-nuclear (ANA) and/or anti-smooth muscle (SMA) antibodies, and AIH-2 being associated with anti-liver kidney microsomal (LKM) and/or anti-liver cytosol type 1 (LC-1) antibodies (5). While AIH-1 affects all ages, AIH-2 affects mainly children and adolescents. Autoimmune sclerosing cholangitis (ASC) is a pediatric condition defined by AIH, almost universally AIH-1, coexisting at diagnosis with abnormal cholangiogram (6). It reportedly affects 40-50% of AIH-1 pediatric patients, and has a more aggressive course, requiring liver transplantation more frequently than classical AIH (6, 7).

Although the aetiology of AIH and ASC remains unknown, genetic and environmental risk factors have been described, the latter including exposure to drugs and viruses (5). While non-HLA polymorphisms have been reported to increase the risk of AIH, the strongest genetic predisposition is conferred by possession of certain HLA alleles: the high diagnostic value of the predisposing HLA alleles is underscored by the inclusion of HLA in the revised AIH diagnostic criteria (5, 8). The HLA predisposing role to AIH, recognized already in 1977 by Opelz et al. and in 1980 by Mackay and Tait, was confirmed in 1991 in a landmark study from King’s College Hospital, London, UK, which identified a significantly higher frequency of the haplotype *A1-B8-DR3* in a cohort including 96 Northern European AIH adolescent and adult patients (three anti-LKM-positive) as compared to healthy controls (9). Possession of this haplotype was also associated to younger age at diagnosis, more frequent relapse and need for liver transplantation (9) (**Table 1**). In DR3-negative subjects, AIH susceptibility was conferred by HLA DR4, which was associated with later disease onset and milder phenotype. Recently, a large paediatric study from the same centre confirmed the association of the *A1-B8-DR3* haplotype with all types of paediatric autoimmune liver diseases, DR3 homozygosity conferring the highest risk, being also associated with fibrosis at disease onset (22). Possession of the HLA-DRB1*03:01 allele and of the A1-B8-DRB1*0301 haplotype is associated with treatment failure and increased risk of cirrhosis, suggesting a more aggressive phenotype in DRB1*03-positive patients (10). Interestingly, this haplotype is linked also to a polymorphism of the *TNF-A* gene, which leads to a higher production of tumour necrosis factor alpha (TNF-α) (10). HLA-DRB1*03 and DRB1*04 encode the amino acid sequences LLEQKR and LLEQRR at positions 67-72 of the HLA β chain (11). HLA-DRB1*1501, which is protective from AIH-1, AIH-2 and ASC in Northern European populations, encodes alanine at position 71, implying that the amino acid at this position is essential to confer susceptibility (22) (**Table 2**).

**Table 1:** Main HLA alleles and haplotypes predisposing to autoimmune hepatitis.

HLA allele or haplotype	Ancestry	Disease	Population age	Reference
*A1-B8-DR3*	Northern Europe	AIH-1 and AIH-2	Adolescent and adults	(5)
*HLA DR4*	Northern Europe, Japan, China, South Korea, India, Iraq, Iran	AIH-1	Adults and children*	(5, 9–17)
A1-B8-DRB1*03:01	Northern Europe	AIH-1, AIH-2 and ASC	Children	(6)
HLA-DRB1*03:01 HLA-DRB1*03	Northern Europe, Thailand, Iraq, Iran, Brazil	AIH-1	Adults and children	(5, 6, 9, 16–19)
HLA-DRB1*07	Northern Europe, Iraq	AIH-2	Children	(6, 16)
HLA-DRB1*08	India, Iran	AIH-1	Adults	(15, 20, 21)
*HLA B*	China	AIH-1	Adults	(22)
HLA-DQA1*01:01	Thailand	AIH-1	Adults	(18)
HLA-DRB1*13	India, Iraq, Northern Europe, Brazil, Iran	AIH-1 and ASC	Adults** and children	(6, 15–17, 19)
HLA-DRB1*13:01	Argentina	AIH-1	Children	(23)
HLA-DRB1*14	India	AIH-2	Adults and children	(15)

*in India, Japan and Iraq.

**in Iran.

AIH-1, type 1 autoimmune hepatitis; AIH-2, type 2 autoimmune hepatitis; ASC, autoimmune sclerosing cholangitis.

**Table 2:** HLA alleles protective from autoimmune hepatitis.

HLA allele or haplotype	Ancestry	Disease	Population age	Reference
HLA-DRB1*11	Iraq, Iran	AIH-1	Adults	(16, 17)
HLA-DRB1*13:02	Argentina	AIH-1	Children	(23)
HLA DRB1*15:01	Northern Europe, Iraq, Iran	AIH-1, AIH-2 and ASC	Children	(6, 16, 17)
HLA DRB1*04	Northern Europe	AIH-1, AIH-2 and ASC	Children	(6)
*HLA DRB5*	Iran	AIH-1	Adults	(17)

AIH-1, type 1 autoimmune hepatitis; AIH-2, type 2 autoimmune hepatitis; ASC, autoimmune sclerosing cholangitis.

The strong association of AIH-1 with HLA polymorphisms has been confirmed by the two genome-wide association studies (GWAS) performed so far in AIH, both including only AIH-1 adult patients (12, 13). The first GWAS, carried out in a Northern European population, identified the class II HLA-DRB1*0301 as the allele with the strongest association with AIH-1, and HLA-DRB1*0401 as the second most strongly AIH-1- associated allele. The second GWAS study, carried out in a Chinese population, identified a single nucletotide polymorphism in the class I HLA-B locus as the strongest association with AIH-1. The study also identified two non-HLA loci associated with AIH-1, i.e. the CD28-CTLA4-ICOS and the SYNPR loci, the first coding for proteins involved in T cell activation, and the latter coding for a protein expressed on synaptic membranes (13).

There are geographical and ethnic differences in HLA allele frequencies, mirrored by differences in HLA associated to AIH in different populations. In Japan, China, South Korea and Taiwan, adult AIH-1 is associated with DRB1*04, DRB1*03 being very rare in the general population, thus potentially explaining the different AIH phenotype in East Asia, which is characterised by later onset and milder disease (14–16, 18, 23) (**Table 1**). However, in Thailand AIH-1 is associated with HLA-DRB1*03:01, and HLA-DQA1*01:01 (19). In India, a study including both children and adults, identified DRB1*04 and DRB1*08 as risk alleles for AIH-1, and HLA-DRB1*04 as risk factor for paediatric AIH, in contrast to its protective role in paediatric Northern European populations, and in line with Japanese data (16, 20). Similarly, a small paediatric study from Iraq, not distinguishing between AIH and ASC, reported DRB1*03, DRB1*04 and DRB1*13 being all associated to AIH-1 (17). DRB1*13 was also found to be associated with paediatric AIH-1 in the above-mentioned study from India, which also found an association of AIH-2 with HLA-DRB1*14 (20). Interestingly, a large study from Argentina found that DRB1*13:01 confers susceptibility for paediatric AIH-1, whereas DRB1*13:02, which differs only in one amino acid, confers protection (24) (**Table 1**).

Genetic studies in AIH-2 have been limited by the rarity of the disease. The largest cohort investigated so far is the above-mentioned study by the King’s College Hospital including only children of European ancestry, which reported an association of AIH-2 with HLA-DRB1*07, whereas HLA-DRB1*15 is protective, as it is for AIH-1 and ASC (22). These results have been confirmed by a smaller study from Iraq (17). The predisposing role of DRB1*07 for AIH-2 has been shown also in Brazil (25). The literature on genetic associations of ASC is also scant, since the differentiation of AIH-1 from ASC is not systematically done in paediatric studies. The association of ASC with DRB1*13 reported in the King’s College Hospital study was confirmed by a small study from Brazil (26); this allele was found to be associated to AIH-2 in Iran (27).

A Japanese study reported a role for class I HLA and killer cell immunoglobulin like receptors (KIR) in association in the predisposition to AIH-1 (15). Natural killer (NK) cells express KIR inhibitory receptors on their surface, which recognize self MHC class I molecules, expressed on all nucleated cells: engagement of these inhibitory receptors protects healthy cells from destruction by NK cells. The investigators, besides confirming that HLA-DRB1*04 confers an increased risk of AIH-1 in Japanese adults, found a strong protective role of the combination KIR3DL1/HLA-B Bw4-80Thr, suggesting a role for NK cells in the pathophysiology of AIH-1 (15).

Attempts have been made to investigate the link between HLA alleles and AIH-1 sub-phenotypes, besides the mentioned association of *DRB3* with more severe disease. According to a retrospective Japanese study, the frequency of DRB1*04 is not different in adult AIH-1 patients presenting with acute severe disease as compared to those presenting with chronic AIH (14). Similar results have been reported by a small Greek study (28). A large German study reported that absence of HLA B8 and presence of HLA DR7 are associated with an increased risk of AIH-induced acute liver failure (29).

### Primary biliary cholangitis

PBC, previously referred to as primary biliary cirrhosis, is an uncommon autoimmune liver disease characterized by inflammation and destruction of the small- and medium-sized intrahepatic bile ducts (21). It affects mainly middle-aged women and is associated with anti-mitochondrial antibody and/or PBC-specific ANA, i.e. ANA displaying a rim-like or a multiple dots nuclear staining pattern on HEp2 cells. Similarly to AIH, despite unknown aetiology, predisposing genetic background and environmental triggers have been reported (21, 30). The important predisposition conferred by genetics is reflected by the high PBC concordance rate of 63% in identical twins, being the highest among a host of autoimmune diseases (31). The unique genealogic database of Iceland offered the opportunity to investigate the familial risk of PBC: Ornolfsson et al. not only did confirm an increased risk in first-degree relatives, but also showed that the risk is increased up to fifth-degree relatives (32). Owing to the strong autoimmune features of PBC, HLA polymorphisms have been investigated, leading to the demonstration that DRB1*08 confers predisposition across different populations (33, 34). In a study including a population from the UK and one from Northern Italy, DRB1*08:01, was associated with increased PBC risk, whereas DRB1*13 was protective; DRB1*11 was protective only in the Italian population (35, 36). In China and Japan, the strongest predisposition has been linked to possession of the DRB1*08:03, which is only one amino acid different from DRB1*08:01 and may have a similar role in antigen presentation (37–39). DRB1*11 (in Italy and Japan but not in the UK) and DRB1*13 (in the UK, Northern Italy and Japan) are protective (35, 38, 40). In Japan, HLA-DQB1*06:04 and HLA-DQB1*03:01 have been reported to be protective, the latter being protective also in China, Europe and North America (37, 39, 41, 42). Due to linkage disequilibrium with HLA-DRB1*08:03, DQA1*01:03 and DQB1*06:01 have also been associated to PBC in Japan and China (38, 39). A distinctive HLA association with the haplotype DRB1*0301-DQB1*02:01 has been reported in Sardinia, known for its genetic isolation (43). Associations have been reported also for HLA DP alleles, including HLA DPB1*03:01 in a German study and DPB1*17:01 in a Chinese study (37, 44).

The first GWAS in PBC published in 2009 and performed in a Nort American population, identified *HLA-DQB1* has having the strongest association (45). All subsequent GWAS confirmed the prominent association of HLA with PBC (42, 46–54).

### Primary sclerosing cholangitis

PSC is a rare cholestatic liver disease leading to fibrosis and strictures of the bile ducts, associated with inflammatory bowel disease (IBD) in 80% of patients of Northern European origin, and showing a male preponderance (55). IBD comorbidity is lower in Southern European and Asiatic subjects (55). Affected patients are at increased risk of cholangiocarcinoma, colorectal cancer, gallbladder carcinoma and hepatocellular carcinoma (56).

Although the cause of PSC remains unknown, there is a genetic predisposition, mirrored by a nearly 10-times higher disease risk in first-degree relatives of affected patients (55). Similarly to AIH and PBC, the strongest genetic predisposition is conferred by HLA alleles, i.e. DRB1*03:01 and B*08:01, both being part of the above-mentioned 8.1 ancestral phenotype, which is associated with a host of autoimmune diseases, including AIH and ASC (2, 57–59). A European study, later confirmed by a study including European Americans, Hispanics and African Americans, identified also DRB1*13 as a risk allele, which is included in the haplotype HLA-DRB1*13:01-DQA1*01:03-DQB1*06:03 (60, 61). Since HLA-DRB1*03:01 is also associated to AIH and ASC, and HLA-DRB1*13 is also associated to ASC, these alleles predispose to AIH, ASC and PSC: whether PSC represents a late stage phenotype of ASC, remains an open question; of note, both diseases lack female preponderance (62). However, the HLA-DRB1*15:01, reported to represent a risk allele in PSC (60), was found to be protective to all forms of paediatric autoimmune diseases in an ethnically similar population, suggesting that HLA polymorphisms are only a component of the complex etiopathogenesis of AIH, ASC and PSC.

The PSC-associated haplotypes A*01-C*07-B*08-DRB1*0301-DQB1*0201 and A*03-C*07-B*07 -DRB1*1501-DQB1*0602 share class I antigens with the same binding properties to KIR: these haplotypes do not carry the *HLA-Bw4* and *HLA-C2* alleles, which are ligands for the inhibitory KIRs 3DL1 and 2DL1 (63). This observation suggests a role for NK cells in the pathophysiology of PSC (57).

The GWAS studies carried out so far in PSC confirm that HLA has the strongest association with PSC (55, 56). This observation supports the notion that PSC is an autoimmune disease, despite the paradox of lack of response to immunosuppression, a feature shared with the other cholestatic autoimmune liver disease, namely PBC.

Main HLA alleles and haplotypes predisposing to or protecting from PBC and PSC are summarized in **Table 3**.

**Table 3:** Main HLA alleles and haplotypes predisposing to or protecting from primary biliary cholangitis and primary sclerosing cholangitis.

Predisposing HLA allele or haplotype	Ancestry	Disease	Reference
HLA-DRB1*08	Italy	PBC	(34)
HLA-DRB1*08:01	UK, Italy	PBC	(35)
HLA-DRB1*08:03	China, Japan	PBC	(37, 38)
HLA-DQB1*06:01	China, Japan	PBC	(39, 40)
HLA-DRB1*08:03- HLA-DQB1*06:01	Japan	PBC	(40)
HLA-DRB1*03	Europe	PSC	(58, 60)
HLA-B*08:01	Europe	PSC	(59)
HLA-DRB1*13	Europe, European Americans, African Americans, Hispanics	PSC	(60, 61)
HLA-DRB1*15:01	Europe	PSC	(60)
*HLA-DQB1:02*	Europe	PSC	(60)
HLA-DQA1*05:01	Europe	PSC	(60)
**Protective HLA alleles**			
HLA-DRB1*13	UK, Italy, Japan	PBC	(35)
HLA-DRB1*11	Italy, Japan	PBC	(35, 40)
HLA-DQB1*03:01	Japan, China, Europe, North America	PBC	(35, 39, 40)
HLA-DQB1*06:04	Japan	PBC	(40)
DRB1*13:02-DQB1*06:04	Japan	PBC	(40)
HLA-DRB1*04	Europe, European Americans, African Americans, Hispanics	PSC	(60, 61)
HLA-DQB1*03:02	Europe	PSC	(60)

PBC, primary biliary cholangitis; PSC, primary sclerosing cholangitis.

### Potential treatment implications

Though the predisposition conferred by HLA points towards a prominent pathophysiological role of the adaptive immune system in autoimmune liver diseases, the target antigens of the autoimmune attack in PSC, AIH-1 and ASC remain largely unknown. A precise knowledge of the antigenic peptides binding to the HLA proteins of an individual patient would pave the way to peptide-based therapies, by using modified peptides which block autoreactive T cells upon binding to the HLA groove, forming an HLA peptide inhibitory complex. Therefore, future research should focus on identifying autoantigenic proteins and their immunodominant epitopes.

Whether HLA can be included in an interactome-based approach to identify new drugs needs to be investigated (54, 64).

## Gut microbiome

The human microbiome has been implicated in the occurrence of several immune-mediated diseases, ranging from rheumatoid arthritis, type 1 diabetes, IBD, and multiple sclerosis (65). Intestinal microbiome may be involved also in the pathogenesis of autoimmune liver disease (66, 67). The blood supply to the liver derives in large amount from the splanchnic circulation, making it exposed to bacteria and bacterial products from the intestinal microbiome. Alterations in the intestinal microbiome might lead to liver damage.

### Autoimmune hepatitis

Changes in the intestinal microbiome have been documented in an experimental model of AIH (1) employing HLA-DR3 transgenic mice on the non-obese diabetic (NOD) background. AIH was induced by immunization with a DNA plasmid coding for a fusion protein of human CYP2D6/FTCD, the autoantigens targeted in AIH-2. HLA-DR3, as mentioned above, is strongly linked to AIH. Not only the HLA-DR3 positive mice that were immunized did develop chronic liver injury recapitulating AIH, but they also had a different microbiome composition compared to the HLA-DR3 negative mice. Mice that developed AIH displayed a reduced diversity of their microbiota. Another experimental model using Tet2^ΔDVAV^ mice, which are deficient in the epigenetic regulator Tet methylcytosine dioxygenase 2 (TET2), shows a key role of microbiome and cytotoxic T lymphocytes in inducing a liver disease closely resembling AIH (68). Moreover, a compound of 15 probiotics has been reported to decrease hepatic inflammation, serum transaminase levels, Th1 and Th17 cells, and increase the number of regulatory T cells in a murine model of AIH, while protecting intestinal barrier integrity, blocking lipopolysaccharide (LPS) translocation, inhibiting the toll-like receptor 4/nuclear factor κB (TLR4/NF-κB) pathway activation and the production of inflammatory cytokines in both liver and ileum (69).

Also in humans, AIH appears to be associated with dysbiosis, increased gut permeability, and translocation of intestinal microbial products into the systemic circulation (70). Changes in the microbiome in association to a leakier mucosal barrier might permit translocation of bacteria and their products promoting an inflammatory response. Moreover, molecular mimicry between microbial and host antigens may trigger or intensify an autoaggressive response in genetically-predisposed individuals (71, 72).

Zona occludens 1 and occludin, structural proteins that function in the binding of intestinal epithelial cells, are decreased in patients with AIH compared to health (70), and this decrease worsens with progression of liver disease. Moreover, AIH patients display a reduced number of anaerobes (*bifidobacterium and lactobacillus species*) within the enteric microbiome. The concept of bacterial translocation is supported by the observed increase in plasma levels of lipo-polysaccharide (LPS) in AIH (70), which worsens with more advanced liver damage. Plasma LPS produced from bacterial translocation can reach the liver through the portal vein. LPS binds to toll-like receptors (TLR) on liver cells, including hepatocytes, stellate cells, Kupffer cells, and sinusoidal epithelial cells, leading to their activation and resulting in an inflammatory milieu predisposing to autoimmunity and fibrosis. The bacterial colonization may also favour the secretion of IL-10 by regulatory T cells (73, 74).

Breech of the intestinal mucosal barrier allows migration of gut-derived bacteria and bacterial products from the intestinal lumen to extra-intestinal sites including the liver. Once the translocated bacterial products, including LPS and unmethylated cytosine–phosphate–guanine (CpG), potent stimulators of the innate immune system, reach the liver they activate TLRs and non-obese diabetes (NOD)-like receptors (NLRs). Migration of activated T lymphocytes from the gut to the liver, including those expressing gut-specific homing receptor alpha4beta7-integrin, has also been implicated in the pathogenesis of autoimmune liver damage within a ‘gut/liver axis’ framework (75), though they can also be found in other forms of liver disease (76).

### Primary biliary cholangitis

In PBC an altered enteric microbiome may affect intestinal motility, immunological function and bile secretion (77–79). PBC patients have fewer *Acidobacteria, Lachnobacterium, Bacteroides*, and *Ruminococcus species* than healthy individuals and a higher number of opportunistic microbes, such as *Y-Proteobacteria, Enterobacteriaceae, Neisseriaceae, Spirochaetaceae, Veillonella, Streptococcus, Klebsiella*, and *Actinol species*. In addition, PBC patients have elevated plasma levels of a variety of cytokines including IL-1β, IL-6,IL-8, IL-18, IL-16, IP-10, MIG, IL-2RA, TNF-α, and macrophage migration inhibitory factor; the lower the levels of microbes normally present in health the higher the levels of inflammatory cytokines (80).

These findings suggest that alterations within the microbiome in PBC have an impact on immune function. PBC has also been associated with abnormal accumulation of LPS in biliary epithelial cells (81, 82), which might activate host immune response. Polymorphisms in the gene coding for TLR 4, which binds to LPS, have been demonstrated in PBC and might favour LPS accumulation in biliary epithelial cells (BEC) triggering host immune responses, further supporting a link between microbial products, the immune system, and PBC (83–85). In addition to LPS, other intestinal microbial components, such as flagellin and cytosine-phosphorothioate-guanine oligonucleotide, concur in eliciting BEC-destructive immune responses (65).

Several studies have investigated the role of infectious agents as triggers of the immune attack on BEC in PBC (86). Anti-mitochondrial antibodies exhibit cross-reactivity with antigens of *Escherichia coli* and *Novosphingobium aromaticivorans* (87, 88). *Escherichia coli* or *N. aromaticivorans* infection might provoke, in genetically susceptible individuals, the production of anti-mitochondrial antibodies that target pyruvate dehydrogenase complex-E2 (PDC-E2) through a process of molecular mimicry (83, 87, 89–92). This theory is supported by an animal model where mice inoculated with *N. Aromaticivorans* develop PBC-like disease (93), and by the observation that PDC-E2 shares sequence homologies with intestinal *Escherichia coli* (83, 87, 89–92). Recently, it has been shown that exposure to *Escherichia coli* elicits specific antibody to *Escherichia coli* PDC-E2 that leads to epitope spreading and production of the classical human anti-PDC-E2 autoantibody, possibly being the first step to development of human PBC (30).

In addition to *Escherichia coli, Klebsiella species* have been implicated in the development of PBC through a mechanism of molecular mimicry (94).

### Primary sclerosing cholangitis

The frequent association of PSC with IBD strengthen the notion of a link between the gut microbiome and the liver. Owing to intestinal inflammation allowing translocation of enteric pathogens through the mucosal barrier, enteric microbial components may reach the liver through the portal circulation and provoke damage to cholangiocytes (95). Alternatively, gut activated T cells may home on to the liver and trigger immune-mediated damage (75, 96). However, as mentioned above, presence of gut-derived activated T cells is not confined to PSC and other autoimmune liver diseases (76).

Patients with PSC have a gut microbial profile different from health as well as from patients with IBD without PSC (97–99). PSC patients have a reduction of intestinal bacterial diversity, with an increased presence of *Veillonella, Rothia, Enterococcus, Streptococcus, Clostridium, Lactobacillus, Fusobacterium and Haemophilus species*. In particular, *Enterococcus, Lactobacillus*, and *Fusobacterium species* are associated with PSC independently of IBD, probiotic use, cirrhosis, liver transplantation, ursodeoxycholic acid or antibiotic treatment (97–99).

The use of antibiotics, including vancomycin, metronidazole, tetracycline, sulfasalazine, azithromycin, and minocycline has been reported to improve laboratory indices in PSC, but not the progression of liver disease (100). The improvement observed with the use of antibiotics may derive from modifications in the microbiome that results in a decreased production of inflammatory molecules.

Faecal microbiota transplantation (FMT), which alters the host microbiome, has been attempted as a therapeutic measure for PSC in a small pilot study. Microbial diversity increased and the alkaline phosphatase levels decreased in treated patients, though it is unclear whether this was associated with a clinical benefit (101). In this pilot study FMT was applied through the colonic route, but a small bowel route might be more appropriate based on animal studies showing that small bowel, but not colonic, dysbiosis is associated with hepatobiliary inflammation, and that small bowel bacterial overgrowth results in a PSC-like disease (102). Larger clinical trials are needed to investigate the role of FMT in PSC.

Both PSC and, to a lesser extent, AIH are associated with positivity for the autoantibody referred to as atypical peripheral anti-neutrophil cytoplasmic antibody (pANCA), also known as peripheral anti-nuclear neutrophil antibody (pANNA). This antibody is reportedly directed to Beta-tubulin and cross-reacts with a bacterial antigen, FtsZ (103), an evolutionary precursor protein of Beta-tubulin present in almost all intestinal bacteria, suggesting that molecular mimicry to bacterial products may also play a role in the development of PSC and AIH.

## Conclusions

The strongest genetic predisposition to all autoimmune liver diseases is conferred by HLA alleles, suggesting a preponderant role of adaptive immunity and particularly of T cells in their pathophysiology. Therefore, knowledge of the autoantigens recognized by T cells is key to advance our understanding of the immunopathogenesis of these diseases. Even in the diseases where the autoantigens are known (AIH-2 and PBC), little is known about the T cells autoantigenic peptides, knowledge of which could pave the way to novel therapeutic approaches. The role of the intestinal microbiome in shaping the phenotype of autoimmune liver disease is currently under investigation. Though most microbiome studies were conducted in wealthy countries in North America and Europe, skewing our understanding of human-microbe interactions, preliminary findings highlight the potential importance of its manipulation in therapeutic interventions.

## Author contributions

BTB-P drafting the first part of the manuscript, critical review and approval of final version. GM-V, DV: drafting the second part of the manuscript, critical review and approval of final version. All authors contributed to the article and approved the submitted version.

## Conflict of interest

The authors declare that the research was conducted in the absence of any commercial or financial relationships that could be construed as a potential conflict of interest.

## Publisher’s note

All claims expressed in this article are solely those of the authors and do not necessarily represent those of their affiliated organizations, or those of the publisher, the editors and the reviewers. Any product that may be evaluated in this article, or claim that may be made by its manufacturer, is not guaranteed or endorsed by the publisher.
